# Effect of Gentrification and Residential Mobility on Associations Between Historical Redlining and Life Expectancy at Birth

**DOI:** 10.1007/s11524-025-01041-3

**Published:** 2026-01-03

**Authors:** Daniel Wiese, Jordan Baeker Bispo, Ann C. Klassen, Charnita Zeigler-Johnson, Ahmedin Jemal, Kevin A. Henry, Farhad Islami

**Affiliations:** 1 Department of Surveillance, Prevention, & Health Services Research, American Cancer Society, 270 Peachtree Street NW Suite 1300, Atlanta, GA 30303 USA; 2 Department of Geography, Temple University, 1115 Polett Walk, Philadelphia, PA 19122 USA; 3 Cancer Prevention and Control, Fox Chase Cancer Center, 333 Cottman Ave, Philadelphia, PA 19111 USA; 4 Dornsife School of Public Health, Drexel University, 3215 Market St, Philadelphia, PA 19104 USA

**Keywords:** Redlining, Gentrification, Residential mobility, Socio-spatial mobility, Life expectancy

## Abstract

Historical redlining, a housing policy applied across numerous cities in the United States in the 1930s, resulted in disinvestment in predominantly non-White, immigrant, or impoverished neighborhoods and has been associated with various adverse health outcomes. However, little is known about the effects of gentrification and residential mobility on health in historically redlined neighborhoods. Using linear regression, we evaluated the association between redlining, gentrification, and life expectancy at birth before and after adjustment for socio-spatial residential mobility in the Philadelphia metropolitan area. Tract-level data on life expectancy were obtained from the Centers for Disease Control and Prevention. Census data were used to define tract gentrification status in 2000–2010 (earlier-gentrification) and 2011–2018 (recent-gentrification). Tract socio-spatial mobility characteristics were defined using DataAxle’s household-level data. Life expectancy at birth was generally higher in historically privileged than disadvantaged tracts. After stratification by gentrification status, however, life expectancy in historically disadvantaged (redlined) areas was higher in earlier-gentrified than non-gentrified tracts. This difference was largely explained by socio-spatial mobility, namely a greater influx of moderate- and higher-income households to gentrified and lower-income households to non-gentrified tracts. In redlined tracts, for example, life expectancy in the unadjusted model was 4.7 years lower in non-gentrified than earlier-gentrified tracts (confidence interval: − 6.4, − 2.9); this difference decreased to 1.7 years (− 3.2, − 0.1) after adjustment for socio-spatial mobility. These findings have substantial implications for future public health research, highlighting the importance of including gentrification and/or socio-spatial mobility data when evaluating the association between historical policies, like redlining, and health outcomes in a given geographic area.

**Supplementary Information:**

The online version contains supplementary material available at 10.1007/s11524-025-01041-3.

## Introduction

The Home Owners’ Loan Corporation (HOLC) housing policy, commonly known as “redlining,” was applied in numerous cities in the United States (U.S.) in the 1930s to stabilize housing market following the Great Depression. To implement this policy, the HOLC developed color-coded ‘residential security’ maps, which graded urban neighborhoods from A/“best” (green) to D/“hazardous” (red) [1] to stimulate or warn against investment. While HOLC maps were drawn upon the existing residential segregation practices of that time [2], residents’ ethnicity and socio-economic status (SES) were among the major factors in designing the grading system [3]. As a result, investments and mortgage expansion were mostly granted to residents in “best”, predominantly wealthy and White neighborhoods, and rarely to residents in “hazardous” neighborhoods, which were predominantly inhabited by Black, ethnically minoritized populations (e.g., Jewish and Eastern/Southern European), immigrant, and impoverished White communities. The “hazardous” neighborhoods are also known as “redlined” areas due to the designated color on HOLC maps. Although this policy was illegal starting in 1968, it contributed to persistent segregation, disinvestment, and high poverty rates and has been associated with adverse health outcomes, including shorter life expectancy at birth, in historically redlined areas [4–8].

A limitation of previous studies on health outcomes by redlining, however, is that they generally do not account for neighborhood socio-demographic changes since the implementation of that policy [9]. Neighborhoods and their demographic structure can change over time in response to processes like decay of housing stock as well as immigration, residential mobility, and gentrification. Gentrification can be defined as a neighborhood socio-demographic transformation process that typically occurs alongside profit-driven housing modernization and construction in historically disinvested communities. This process generally attracts new residents with higher incomes, often contributing to an increase in the area’s SES, housing value, and rental costs, forcing the existing residents to relocate to more affordable neighborhoods [10]. Although gentrification may positively affect neighborhoods by improving the built environment, its effect on population health remains largely unknown because of the small amount of research and inconsistent findings. For example, a study of the 500 largest U.S. cities using aggregated data reports a significant positive association between gentrification and self-rated health [11]. In contrast, studies using individual-level data have found no overall association between gentrification and individuals’ self-rated health status, but a significant inverse association among Black individuals or individuals of lower income levels (defined as income level at various thresholds below the federal poverty line) [12–14]. These inconsistent findings may be partly or entirely due to past research's failure to account for socio-spatial residential mobility, which describes a relationship between an individual’s geographic relocation and potential changes in the area-level SES. In a gentrifying area, this would suggest a replacement of residents of lower income levels with newcomers of higher income levels.

To address the research gap on the potential effects of gentrification on health outcomes associated with historical redlining, we aim to demonstrate the importance of accounting for gentrification and residential mobility when examining the association between historical redlining and life expectancy at birth. We hypothesize that the life expectancy at birth does vary by neighborhood gentrification status, specifically within the historically redlined areas, and this difference is related to the duration of the gentrification process and the intensity of the population mobility. This study focuses on the Philadelphia metropolitan area from 2011 to 2018, but findings may inform broader investigations of historical policy impacts on contemporary health outcomes. Philadelphia has been a subject in earlier studies on the consequences of redlining [15] and gentrification [16] on contemporary health outcomes and is known for existing health disparities between the neighborhoods [17–19]. Integrating residential mobility into our study, we aim to contribute to the literature on finding the potential explanatory factors for the existing difference in life expectancy at birth and potentially other health outcomes in the context of historical redlining and gentrification.

## Methods

### Study Design

In this ecological cross-sectional study, we aimed to examine the association between historical redlining, gentrification, and life expectancy at birth before and after adjustment for socio-spatial residential mobility in the Philadelphia metropolitan area in 2011–2018. The goal of this study was to demonstrate the importance of accounting for gentrification and residential mobility when examining the association between a historical policy (e.g., redlining) and a health outcome (e.g., life expectancy at birth). This study was motivated by prior discourse on how to account for socio-demographic and economic shifts occurring in historically redlined neighborhoods experiencing increased capital investment (e.g., gentrification), and how these changes may affect contemporary population health outcomes [9, 15]. Analysis was conducted at the census tract level (*n* = 1457), using aggregated household-level data (*n* = 2.8 million). This study was exempt from institutional review, and informed consent was waived according to the Common Rule (45 CFR §46), as the analysis of deidentified, publicly available data is not considered human participant research.

### Life Expectancy at Birth

Life expectancy at birth is an average estimate of expected years to live from birth for each individual in a designated area (i.e., country, state), based on recent age-group-specific mortality rates [20]. Because of its correlation with several health outcomes and health-related risk factors, life expectancy can be seen as a summary indicator of population health/well-being; often used to evaluate public health interventions [21]. The most recent census tract-based estimates were obtained from the Centers for Disease Control and Prevention (CDC) [22].

### Census Tract Categorization Based on HOLC Grade and Gentrification Status

Historical redlining data, based on the 1930s neighborhood boundaries and transformed to corresponding modern tract borders, were obtained from the Interuniversity Consortium for Political and Social Research [23]. Their methodology included adaptation of the historical HOLC grades to modern-day census tract borders. Particularly, if a tract included different HOLC grades (e.g., “definitely declining” and “hazardous”), the category was assigned based on the largest proportion of that given grade. Tracts were classified according to the following HOLC grades: “best,” “still desirable,” “definitely declining,” and “hazardous.” If a tract was not assigned any grade (i.e., because it was not considered urban when HOLC maps were produced), it was categorized as “unclassified”.

To define the gentrification status of each tract, we adapted the methodology established by Sutton [24]. We created a gentrification index using a weighted composite score derived from the sum of percentage point differences within a multi-year period on three underlying socio-demographic attributes. These were the proportion of college-educated residents, the proportion of neighborhood short-tenured population (< 5 years), and the proportion of owner-occupied housing units.

In this study, we evaluated two time periods for gentrification: 2000–2010 and 2011–2018. Gentrification categories were defined as “ineligible for gentrification” if a tract’s median household income level was ≥ 40% above the metropolitan area average as of 2011; “earlier gentrification” if a tract gentrified in 2000–2010; “recent gentrification” if a tract gentrified in 2011–2018; and “no gentrification” if a tract was eligible to gentrify in 2011 but did not experience any substantial transformation (< 1 standard deviation of the metropolitan area’s average). All sociodemographic characteristics were based on the U.S. Census 2000 and American Community Survey 2006–2010, 2007–2011, and 2014–2018 estimates [25].

### Census Tract Socio-Spatial Residential Mobility

Socio-spatial residential mobility describes a relationship between an individual’s geographic relocation and potential changes in the SES or income level and can then be used as an indicator of neighborhood socio-demographic transformation [26]. We characterized tract-level socio-spatial mobility patterns by aggregating household-level data from the DataAxle company, a major consumer trace data provider for marketing research. DataAxle’s database is compiled from various sources, including real estate, tax assessments, and others [27]. In this study, we used annual information about the primary residential location and income of households within the study area. A previous study on population mobility in the U.S. has reported that DataAxle household data have high quality and are representative of the U.S. population [28].

First, we examined the out-moving population by extracting every household with a primary residence in the Philadelphia metropolitan area as of 2011 with complete residential history from 2011 through 2018, geocoded to the tract level. This time period was selected to capture the variation in the socio-spatial residential mobility patterns across tracts that gentrified earlier (i.e., advanced or completed gentrification by 2011) and recently (i.e., early stages of gentrification, starting since 2011) because of the expected differences in the income level of the incoming population by area gentrification status [29]. Households with incomplete residential and income information (8.7%) were excluded to avoid a potential attrition bias related to the mover status, as it was unknown whether a household was lost to follow-up because of a relocation or death. For every household, we defined their final destination tract and income level using the most recent year of available data through 2018. Households were considered out-movers if they were no longer residing in their 2011 tract. Next, we examined the in-moving population by tracking back their residential histories from 2018 through 2011 and defined their origin and destination tracts and income level. For example, if a household relocated to a different tract within the Philadelphia metropolitan area, they were coded as out-movers in their 2011 tract and as in-movers in their new tract. If a household was no longer in the database by 2018 or relocated outside of the Philadelphia metropolitan area, they were coded only as out-movers in their 2011 tract. The households that relocated from the outside to inside of the Philadelphia metropolitan area were coded only as in-movers in their destination tract. For the households that moved multiple times throughout the study period, we only considered their first and last locations. Household income categories were defined based on quartiles and included lower-income (lowest quartile, Q1), moderate-income (quartiles Q2–Q3), and higher-income (highest quartile, Q4).

All households (represented in the origin and/or current population; *n* = 2.8 million) were then categorized based on their income level and mobility patterns (Supplemental Table 1) and linked to the tract transformation status (HOLC grade and gentrification status combined) of their origin and destination tracts. For every tract, we then counted the net change in the number of in-moving and out-moving lower-income, moderate-income, and higher-income households and developed three measures of socio-spatial mobility characteristics (net change in lower-income, moderate-income, and higher-income households), each classified according to the magnitude of the change based on quartiles (major increase, minor increase, minor decline, or major decline)—exact numeric definitions are described in Table 1. Lastly, we summarized the number and proportion of tracts with increasing and decreasing numbers of lower-income, moderate-income, and higher-income households by area gentrification status.

**Table 1:** Association between census tract-level socio-spatial mobility characteristics and life expectancy at birth

Socio-spatial mobility characteristics	Unadjusted model
	**Coefficient (CI95%)**
Net change in lower-income households (***n***)	
Major decline (−277 to −18)	Ref
Minor decline (−17 to −1)	− 0.41 (− 0.81 to − 0.02)
Minor increase (+ 1 to + 30)	− 2.17 (− 2.63 to − 1.73)
Major increase (+ 31 to + 380)	− 2.60 (− 3.04 to − 2.17)
Net change in moderate-income households	
Major decline (−732 to −36)	Ref
Minor decline (−35 to −1)	− 0.09 (− 0.57 to 0.35)
Minor increase (+ 1 to + 47)	1.07 (0.62 to 1.54)
Major increase (+ 48 to + 501)	1.25 (0.77 to 1.70)
Net change in higher-income households	
Major decline (−449 to −24)	Ref
Minor decline (−23 to −1)	− 1.62 (− 1.99 to − 1.21)
Minor increase (+ 1 to + 63)	1.20 (0.69 to 1.72)
Major increase (+ 64 to + 658)	2.05 (1.49 to 2.60)

The net change in lower-income, moderate-income, and higher-income number of households (*n*) was classified according to the magnitude of change based on quartiles (major increase, minor increase, minor decline, or major decline)

### Statistical Analysis

The average population-weighted life expectancy and the corresponding 95% confidence interval (95%CI) were estimated for each HOLC grade (five categories) and gentrification status (four categories). Using unadjusted linear regression models, we first examined the association between tract socio-spatial mobility characteristics and life expectancy at birth. We then modeled the association between tract gentrification status and life expectancy before and after the adjustment for socio-spatial mobility characteristics, stratified by HOLC grade. Because tract gentrification status is a composite measure based on educational status and housing tenure, we did not adjust our models for any other socioeconomic factors to avoid collinearity. In a sensitivity analysis, we further adjusted our models for tract-level percent of change in non-White population during 2011–2018.

Lastly, we developed an unstratified spatial Bayesian regression model (R package BayesX) [30] to examine the association between tract HOLC grade, gentrification status (reference group: “unclassified and ineligible for gentrification”), and life expectancy at birth, and to designate tracts with significantly lower or higher life expectancy estimates compared to the metropolitan area average. The spatial function was based on stationary Gaussian random fields (bivariate penalized splines). A P-spline smoothed spatial effect was incorporated based on an adjacency matrix of geographic neighbors (weights based on rook’s case) for each census tract [31]. We were unable to create spatial models stratified by HOLC categories because areas with the same HOLC grade are often disconnected from each other. In this case, stratification of the spatial data results in a failure of the model to integrate the neighborhood continuity matrix and apply spatial interpolation techniques. All analyses were conducted using R statistical software.

## Results

The Philadelphia metropolitan area comprises 11 counties from 4 states (Pennsylvania, New Jersey, Delaware, and Maryland) and includes 1457 census tracts, 55 (3.8%) of which were excluded from the analysis because of missing data on life expectancy or total population. The majority of the remaining tracts (*n* = 987 of 1402, 70.4%) had no HOLC grade (i.e., “unclassified”); 40 (2.9%) tracts were classified as “best,” 131 (9.3%) as “still desirable,” 96 (6.8%) as “definitely declining,” and 148 (10.6%) as “hazardous.” Overall, 316 (22.5%) census tracts experienced either earlier or recent gentrification (Supplemental Table 2). Of 148 historically redlined tracts included in this study area, 65 tracts (43.9%) gentrified either between 2000 and 2010 or 2011 and 2018. Area gentrification status showed substantial variation in socio-spatial mobility patterns. For example, the proportion of tracts with a net increase in the number of higher-income households was highest in areas ineligible for gentrification (81.3%), followed by areas with earlier gentrification (25.5%) and recent gentrification (23.4%), and was lowest in areas with no gentrification (12.0%) (Supplemental Figure 1A). In contrast, the net increase in the number of lower-income households was smallest in areas ineligible for gentrification (3.6%), followed by areas with earlier gentrification (38.3%), recent gentrification (45.5%), and no gentrification (54.2%). Consistent with these findings, the proportion of tracts with declines in the number of households with higher-income levels was largest in areas with no gentrification (88.0%) and lowest in areas ineligible for gentrification (18.7%) (Supplemental Figure 1B).

### Life Expectancy at Birth by HOLC Grade and Gentrification Status

**Fig. 1:**
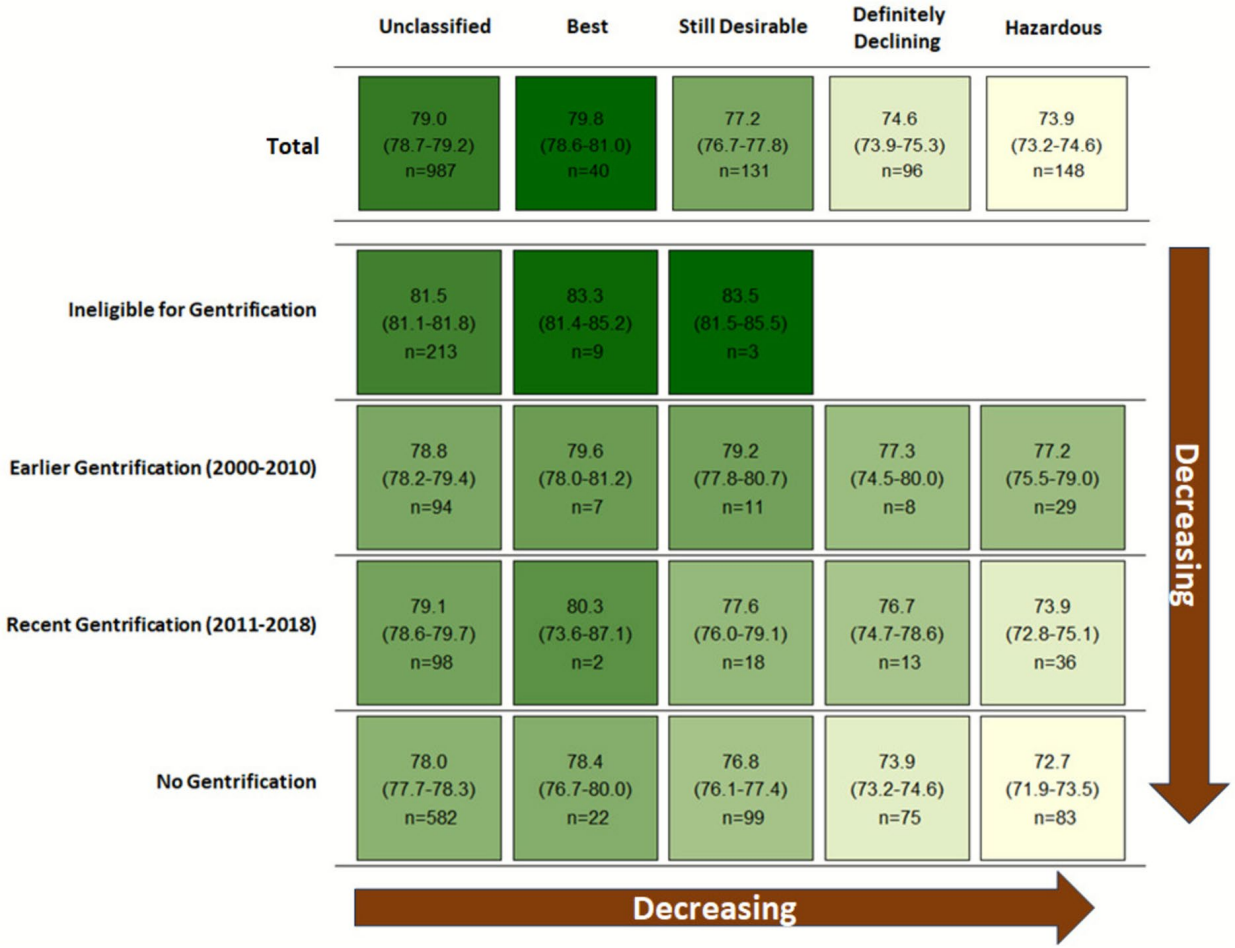
Average population-weighted life expectancy at birth estimates by census tract historical HOLC grade and gentrification status (i.e., transformation status). HOLC, Home Owners’ Loan Corporation. Note: Because all historically “definitely declining” and “hazardous” tracts in the study area were eligible for gentrification, there were no “definitely declining and ineligible for gentrification” and “hazardous and ineligible for gentrification” categories

There was a decreasing gradient in life expectancy from census tracts graded by HOLC as “best” (average = 79.1 year, 95%CI = 77.8 to 80.3), to “still desirable” (77.2, 76.7 to 77.8), to “definitely declining” (74.3, 73.6 to 74.9), and to “hazardous” (73.8, 73.1 to 74.5) (Fig. 1). However, there was substantial heterogeneity in life expectancy across tracts of the same HOLC grade by gentrification status. For example, life expectancy was considerably higher for ‘hazardous’ tracts that experienced earlier gentrification (77.4, 75.8 to 79.1) compared to those that did not gentrify (72.8, 72.0 to 73.6), the latter of which had the lowest life expectancy in this study. The highest average life expectancy estimates were found in census tracts that were ineligible for gentrification because of higher median household incomes, including HOLC “unclassified” (81.5, 81.1 to 81.8) and historically privileged “best” (83.1, 81.4 to 84.8) and “still desirable” (83.6, 81.9 to 85.3) tracts.

### Associations Between Tract-Level Socio-Spatial Mobility Characteristics and Life Expectancy

Compared to tracts with a major decline in the number of lower-income households, life expectancy was lower in tracts with a major increase in lower-income households (regression coefficient = − 2.60; 95%CI = − 3.04 to − 2.17) (Table 1). Conversely, life expectancy was higher in tracts with a major increase in moderate-income level households (1.25; 0.77 to 1.70) or higher-income level households (2.05; 1.49 to 2.60) than in tracts with major declines in these groups.

### Association Between Gentrification Status and Life Expectancy

**Fig. 2:**
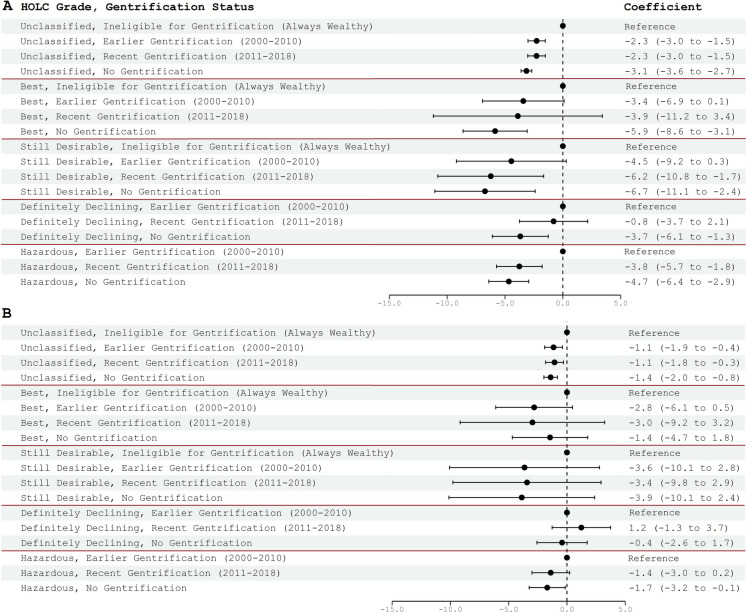
Regression coefficients for the association between census tract gentrification status and life expectancy at birth, stratified by HOLC grade. **A** Unadjusted model; **B** model adjusted for socio-spatial mobility characteristics. HOLC, Home Owners’ Loan Corporation

In unadjusted models, life expectancy at birth across all HOLC grades decreased from tracts ineligible for gentrification or with earlier gentrification to tracts with recent gentrification to non-gentrified tracts (Fig. 2A). Among the “unclassified,” “best,” and “still desirable” areas, life expectancy in non-gentrified tracts was significantly lower compared to tracts ineligible for gentrification, but it was not significantly different from the tracts with earlier or recent gentrification. In areas classified as “definitely declining” and “hazardous,” however, life expectancy was significantly lower in non-gentrified tracts compared to tracts with earlier gentrification, by 3.7 (regression coefficient = −3.7; 95% CI = − 6.1 to − 1.3) and 4.7 (− 4.7; − 6.4 to − 2.9) years, respectively. For the “hazardous” areas, coefficients for the tracts with recent gentrification were more comparable to the non-gentrified tracts than to those with earlier gentrification.

After adjusting for the tract socio-spatial mobility characteristics, regression coefficients attenuated across all HOLC grades (Fig. 2B). There was also no longer a significant difference between tracts with no gentrification and earlier gentrification in ‘definitely declining’ areas (regression coefficient = − 0.4; 95%CI = − 2.6 to 1.7), but the difference persisted—though to a minimal extent—in “hazardous” areas (− 1.7; − 3.2 to − 0.1). Coefficients for the “hazardous” tracts with recent gentrification were not statistically significantly different from “hazardous” tracts with earlier gentrification (− 1.4; − 3.0 to 0.2). In a sensitivity analysis, additional adjustment for tract-level percent of change in non-White population did not affect regression coefficients described above (results not shown).

In the unadjusted spatial model without stratification by HOLC status (Supplemental Figure 2A), the overall patterns in the relation between HOLC grades, gentrification, and life expectancy were comparable to HOLC-stratified models. There was no statistically significant difference in life expectancy between the reference group (unclassified and ineligible for gentrification) and other ineligible for gentrification tracts across all HOLC grades as well as tracts in the category “best” with any gentrification status. Within each HOLC grade, life expectancy decreased from tracts ineligible for gentrification or with earlier gentrification to tracts with recent gentrification to tracts with no gentrification, with larger differences in life expectancy by gentrification status among “definitely declining” and “hazardous” tracts. After adjustments for tract socio-spatial mobility, regression coefficients were generally attenuated (Supplemental Figure 2B). Compared to the “unclassified and ineligible for gentrification” tracts, life expectancy remained significantly lower only in tracts with transformation status “definitely declining and no gentrification” and all three “hazardous” categories irrespective of gentrification status. There was no statistically significant difference between tracts with earlier, recent, or no gentrification in “definitely declining” and “hazardous” HOLC grades.

**Fig. 3:**
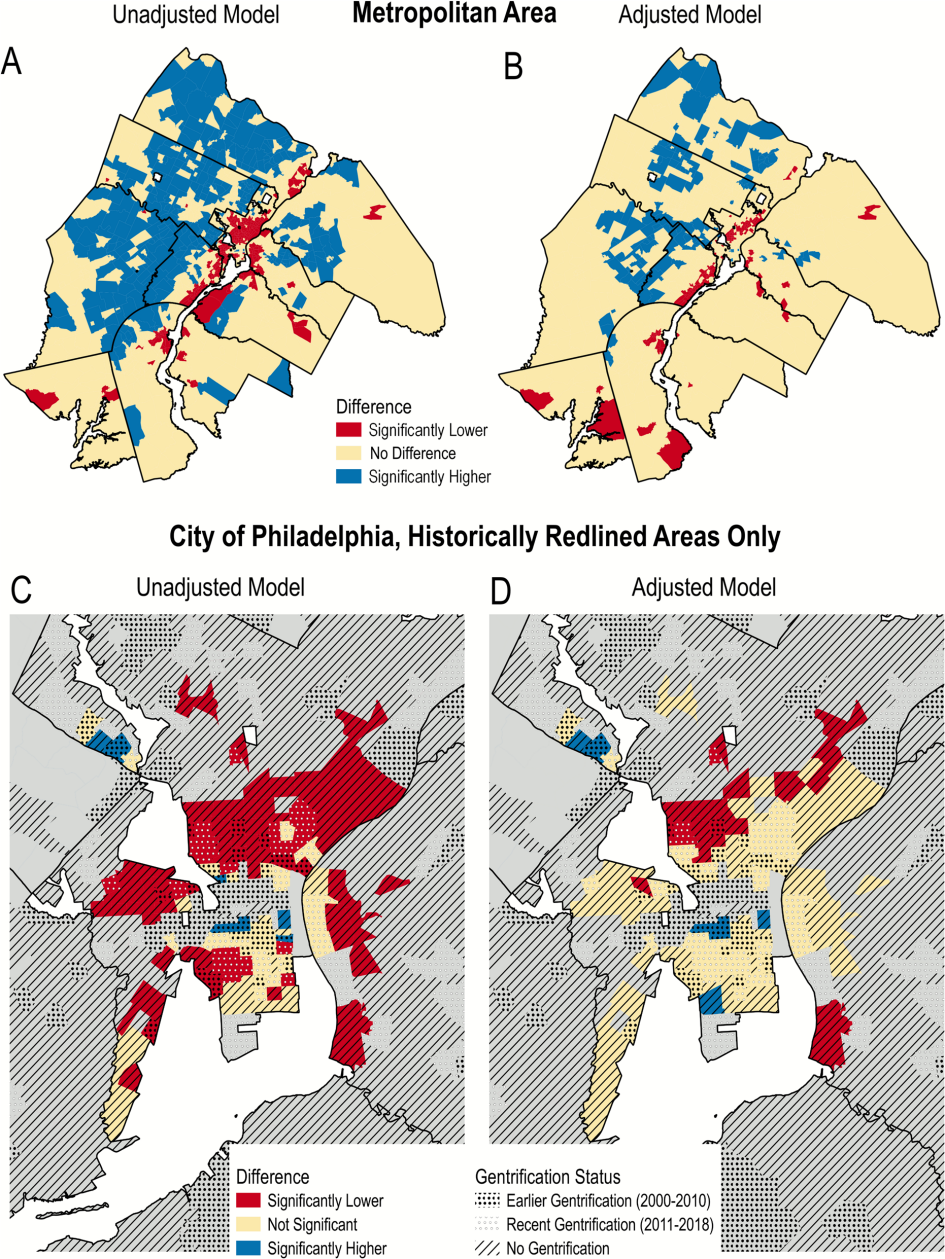
Areas with significantly lower, higher or not significantly different life expectancy estimates compared to the metropolitan area average. Results are based on unadjusted spatial model (**A, C**) and after adjustment for tract socio-spatial mobility characteristics (**B, D**). Note: Statistical significance in the Bayesian spatial model was assessed by whether the 95% credible interval of the posterior distribution excluded zero

Geographically, in unadjusted models (Fig. 3A), tracts with significantly lower life expectancy estimates compared to the metropolitan area average were predominantly found in the City of Philadelphia and several smaller towns along the Delaware River. After the adjustment for tract socio-spatial mobility (Fig. 3B), the number of these tracts was substantially reduced. When focusing on historically redlined areas in the City of Philadelphia only (Fig. 3C, D), we found that after the adjustment for tract socio-spatial mobility, life expectancy in most of the tracts was no longer significantly different from the metropolitan area average; and those tracts that had significantly lower life expectancy were predominantly non-gentrified tracts.

## Discussion

This study is the first to assess the contribution of socio-spatial residential mobility in explaining the association between historical redlining, gentrification, and a health outcome, i.e., life expectancy at birth. Similar to previous research, our results showed that neighborhoods with historically lowest, unfavorable HOLC grades have the lowest average life expectancy [4], likely due to the contribution of that policy to persistent disinvestment in those tracts. However, we found that gentrification is critical when evaluating the differences in life expectancy between tracts of historically unfavorable HOLC grades (e.g., “definitely declining” or “hazardous”/redlined) because the estimates in non-gentrified tracts are significantly lower than those in tracts with earlier gentrification. This difference in life expectancy by gentrification status was largely explained by the socio-spatial mobility patterns, namely the major increases of moderate and higher-income households in gentrified tracts and lower-income households in non-gentrified tracts, as higher income populations are more likely to have higher life expectancy [32]. By demonstrating the effect of gentrification and attributed socio-spatial mobility flows on the described differences in life expectancy between tracts, we support earlier recommendations that the effects of historical policies, like redlining, should not be examined without consideration of contemporary socio-demographic transformation processes [15].

A relatively higher life expectancy in areas with earlier gentrification than recent gentrification is likely due to socio-spatial population mobility after the start of gentrification. In the beginning stages of gentrification, the incoming population (i.e., the pioneers) is often of moderate-income level; this movement is typically followed by an increase in households with higher income, especially with longer durations from the start of gentrification [29]. The remaining relatively small, unexplained difference between historically redlined areas with earlier gentrification and no gentrification could be a result of different factors. First, we may not have fully captured the socio-spatial mobility using DataAxle data. Second, the improved built environment as a result of gentrification might improve health and life expectancy to some degree, although more research on this possible association is needed. Additionally, more attention should be given to the historically “yellow-lined” areas (HOLC category C/“definitely declining”). An earlier study from Philadelphia and Baltimore found that while historically redlined neighborhoods experienced some improvement in the built environment, likely because of gentrification, historically “yellow-lined” areas had the most adverse status of social and environmental determinants of health [33]. Our results show that compared to historically redlined areas, only a small number of “yellow-lined” tracts experienced any gentrification. These “yellow-lined” tracts not affected by gentrification may become potential destinations for lower-income displaced populations but remain largely disinvested, which may influence future population health outcomes.

Our findings have also a broad implication for the discourse on gentrification as a driver of health outcomes and future health equity research. First, the contribution of socio-spatial mobility characteristics in explaining differences in life expectancy estimates between gentrified and non-gentrified tracts suggests that positive associations between gentrification and health outcomes should be interpreted with caution because it underscores the possibility that population change is driving the association. Second, our findings also indicate that gentrification and socio-spatial population mobility could be among the contributing factors explaining differences and temporal trends in other tract-level health outcomes, especially in urban and historically disadvantaged neighborhoods. Lastly, considering the difference in socio-spatial mobility characteristics between neighborhoods, it is reasonable to assume that distressed communities undergoing gentrification experience an influx of capital, resources, and development. However, further studies are needed to examine whether the associated health benefits are impacting the residents in a way that is equitable, as well as health outcomes among displaced lower-income populations that are forced to move out of gentrified areas. The interplay of neighborhood racial dynamics on gentrification, residential mobility, and outcomes like life expectancy must also be further investigated.

Prior research has highlighted adverse associations between gentrification and displacement among marginalized racial/ethnic groups. For example, residents of historically redlined neighborhoods undergoing gentrification or experiencing population displacement are reported to have significantly higher odds of experiencing severe maternal morbidity, especially among Black people, compared with those in continuously privileged neighborhoods [34]. Moreover, both historical redlining and gentrification may present challenges to residents in finding housing that is adequate in quality (housing conditions are often poor in historically redlined areas) and affordable (rent price is typically high in gentrified areas), negatively influencing mental health among Black residents [35]. Especially in a region like Philadelphia with a large non-White population and a legacy of racial segregation, individuals’ race/ethnicity may play a role in the choice of housing. In this study, adjustment for tract-level change in non-White population did not change our results, likely in part due to correlation between race/ethnicity and the socioeconomic factors that were used to measure gentrification and population mobility. However, future studies are warranted that evaluate whether associations between gentrification and health differ according to racial/ethnic characteristics at both neighborhood and individual levels.

Despite the uniqueness of this study, several limitations should be mentioned. First, while the concept of gentrification is well established, there is no standardized methodology to measure gentrification. Using other gentrification indices could result in some variations in the outcomes. However, considering theoretical and methodological similarities between most indices [36], it is unlikely that the results would vary substantially. Second, although household-level residential mobility data used in this study for the definition of socio-spatial mobility characteristics have been shown to be representative of the U.S. population [28], it is not a full sample. We excluded 8.7% of the initial household records because of incomplete residential histories and missing income level information. Additionally, misclassification of the primary residence among households that own multiple homes is possible and the number of mover households, particularly lower-income renters, may be undercounted/underrepresented. Nevertheless, DataAxle provides an opportunity to derive micro-level mobility measures, which cannot be derived using aggregated census data. Third, tract-level life expectancy estimates may differ in the magnitude of uncertainty, often with higher uncertainty in large, rural, and primarily non-residential tracts (such as primarily commercial and industrial tracts) with smaller population sizes. To reduce uncertainty, we excluded all non-residential tracts (*n* = 55); no rural tracts were included in this study by design. Lastly, we were not able to conduct a longitudinal analysis of the effects of socio-spatial residential mobility on changes in life expectancy at birth because tract-level data on life expectancy were available for only one time period. A further analysis of the effects of gentrification and socio-spatial residential mobility on changes in life expectancy estimates (upon the release of these data) is needed. Moreover, we were only able to conduct this study including the data up to 2018 to match the approximate timespan when life expectancy was estimated. However, the effect of the COVID-19 pandemic on the life expectancy estimates as well as on population mobility requires further research.

## Conclusion

The present study is the first to demonstrate the importance of accounting for gentrification and socio-spatial residential mobility patterns when examining the association between historical redlining and a health outcome. Moreover, in contrast to previous research on gentrification, solely relying on aggregated census data, our study includes household-level residential mobility data, which captures the dynamic nature of gentrification. The findings from this study have substantial implications for future public health research, highlighting the importance of including socio-spatial residential mobility data when evaluating the association between historical events or policies, like redlining, and health outcomes in a given geographic area. Additionally, results from this study suggest that future research on gentrification and population health should be examined with care because an improvement in a health outcome at the neighborhood level over time may not always reflect the improvement of health among the initial residents. Rather, this improvement might be driven by the residential mobility and replacement of residents with different socio-economic status characteristics.

## Supplementary Information

Below is the link to the electronic supplementary material.

ESM 1 Supplementary Material 1 (DOCX 512 KB)

## Data Availability

No individual participant-level data were used in this study. Data on household-level residential mobility were purchased from a commercial company, DataAxle. Socio-economic and demographic census tract variables are publicly available from the U.S. Census Bureau.

## Code Availability

The analysis was conducted using R statistical software. The code is available from the corresponding author upon reasonable request.
