# Supplementary material for: Effect of Gentrification and Residential Mobility on Associations Between Historical Redlining and Life Expectancy at Birth
Source: J Urban Health. 2026 Jan 3;103(1):135–46. doi: 10.1007/s11524-025-01041-3 (PMC13136437; doi:10.1007/s11524-025-01041-3)

**Supplementary Materials**

**Supplemental Table 1.** Household categorization based on geographic mobility patterns and income-level.

| Household category | Definition |
| --- | --- |
| Non-movers | Households that remained in the same residential tract during the entire study period. |
| Out-movers | Households that moved from their original 2011 tract to any other location. |
| In-movers | Households that moved/relocated to a tract in Philadelphia metropolitan area by the end of the study period (originating from the inside or outside the metropolitan area). |
| Lower-income in-movers/out-movers | Lower-income households that moved/relocated into or out from any tract in Philadelphia metropolitan area by the end of the study period. |
| Moderate-income in-movers/ out-movers | Moderate-income households that moved/relocated into or out from any tract in Philadelphia metropolitan area by the end of the study period. |
| Higher-income in-movers/ out-movers | Higher-income households that moved/relocated into or out from any tract in Philadelphia metropolitan area by the end of the study period. |

Note: A household can be an out-mover in tract X and an in-mover in tract Y. Non-movers were not considered in this study. Household income categories were defined based on quartiles and included: lower-income (lowest quartile, Q1), moderate-income (quartiles Q2-Q3), and higher-income (highest quartile, Q4).

**Supplemental Table 2.** Distribution of census tracts by historical Home Owners’ Loan Corporation (HOLC) grade and gentrification status.

|  | **Best (N=40)** | **Still Desirable (N=131)** | **Definitely Declining (N=96)** | **Hazardous (N=148)** | **Unclassified (N=987)** | **Total (N=1402)** |
| --- | --- | --- | --- | --- | --- | --- |
| **Gentrification status** |  |  |  |  |  |  |
| Ineligible for Gentrification (Always Wealthy) | 9 (22.5%) | 3 (2.3%) | 0 (0%) | 0 (0%) | 213 (21.6%) | 225 (16.0%) |
| Earlier Gentrification (2000-2010) | 7 (17.5%) | 11 (8.4%) | 8 (8.3%) | 29 (19.6%) | 94 (9.5%) | 149 (10.6%) |
| Recent Gentrification (2011-2018) | 2 (5.0%) | 18 (13.7%) | 13 (13.5%) | 36 (24.3%) | 98 (9.9%) | 167 (11.9%) |
| No Gentrification | 22 (55.0%) | 99 (75.6%) | 75 (78.1%) | 83 (56.1%) | 582 (59.0%) | 861 (61.4%) |

**Supplemental Figure 1.** Socio-spatial mobility patterns by census tract gentrification status. Figure A shows the proportion of census tracts with a net increase in the number of households of different income levels. Figure B shows the proportion of census tract with a net decrease in the number of households of different income levels.


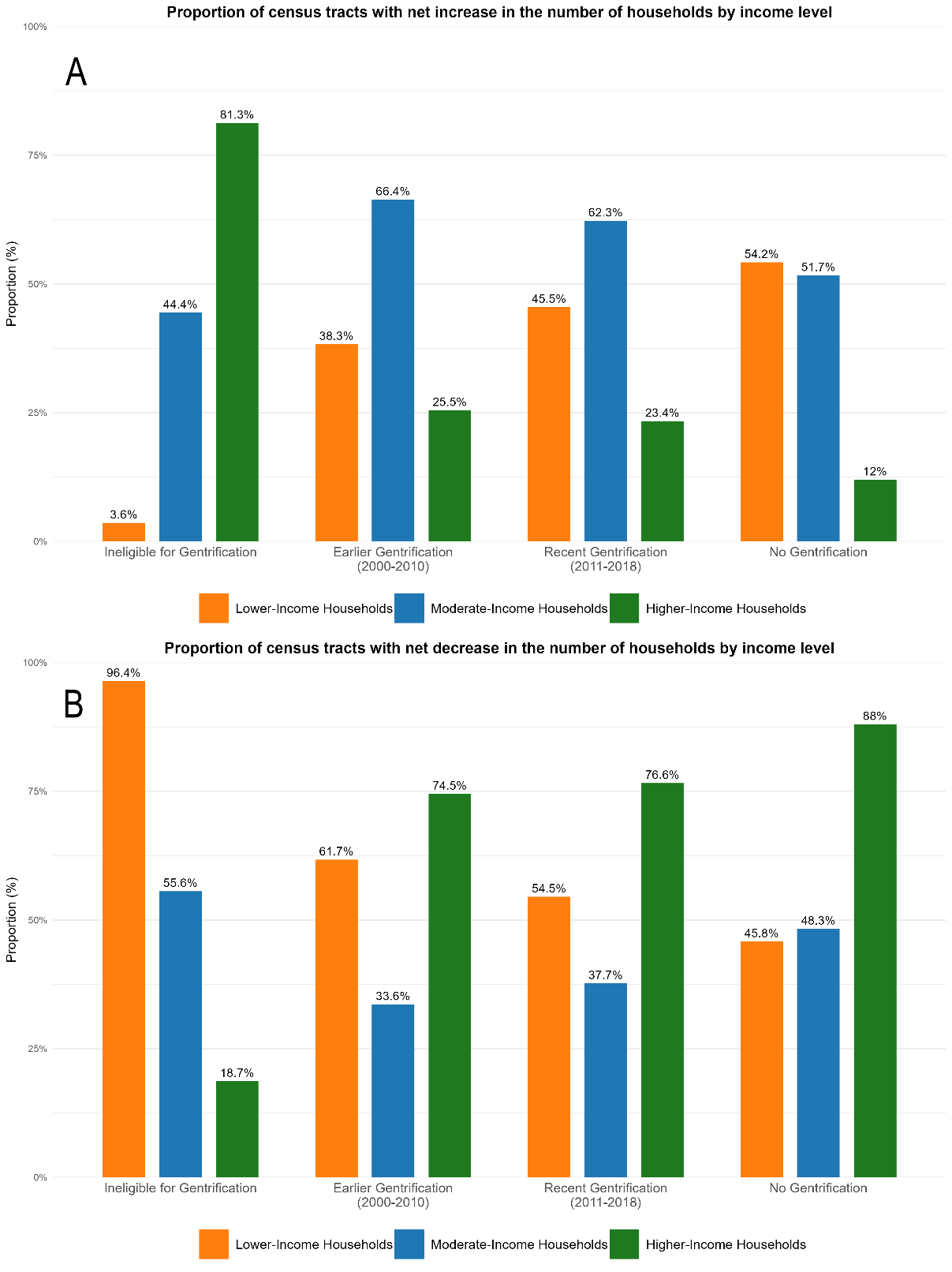


Note: Proportions of tracts do not add to 100% because a tract can experience a net increase and/or decrease in the number of households of all income levels.

**Supplemental Figure 2.** Regression coefficients for the association between census tract transformation status (HOLC grade and gentrification status combined) and life expectancy at birth. A: spatial unadjusted model; B: spatial model adjusted for socio-spatial mobility characteristics. HOLC, Home Owners’ Loan Corporation.


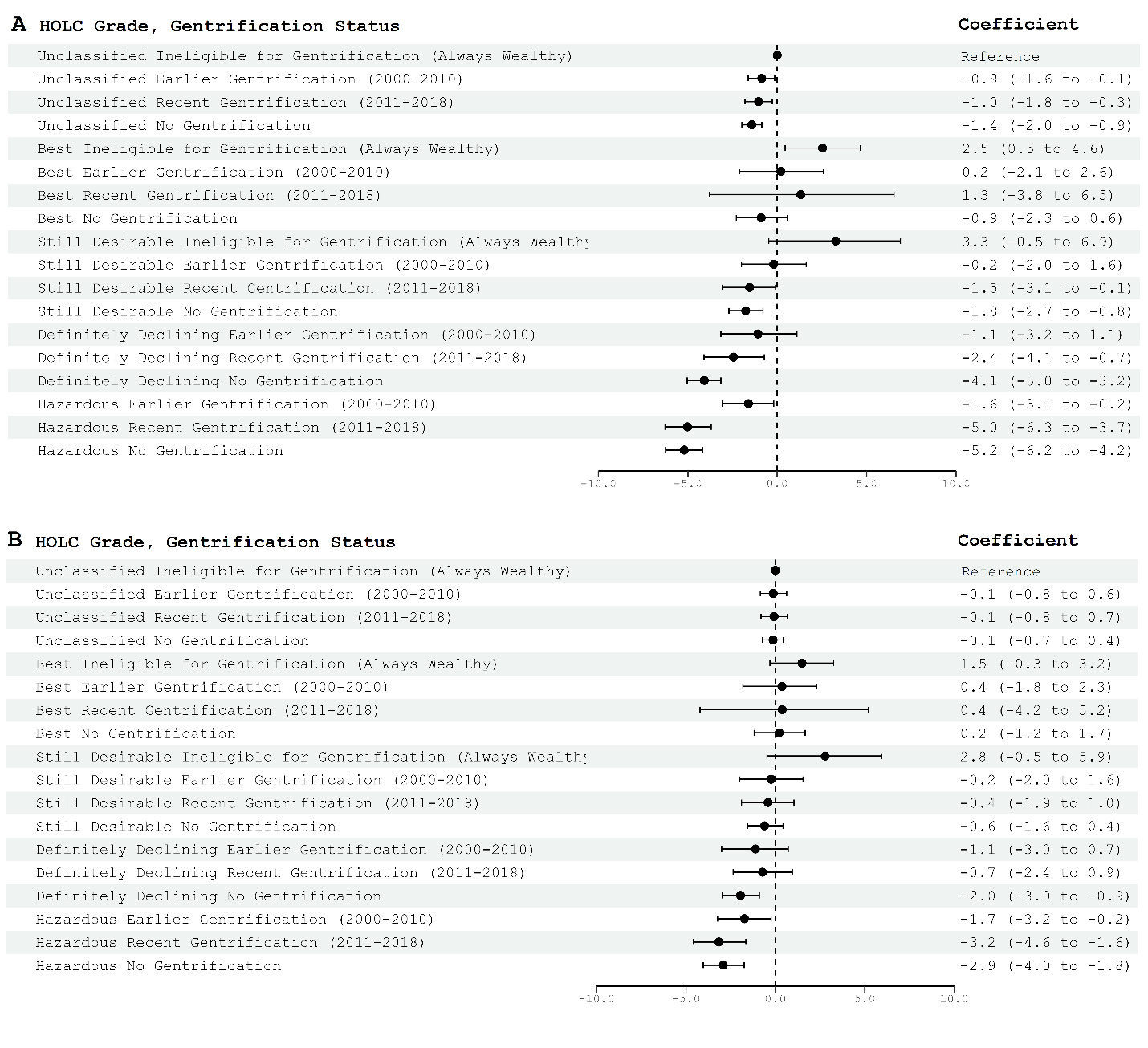

Supplement: Supplementary file 1 — Supplementary Material 1 (DOCX 512 KB) [file 11524_2025_1041_MOESM1_ESM.docx]
